# Host Preference of *Bactrocera latifrons* (Hendel) (Diptera: Tephritidae) Among Fruits of Solanaceous Plants

**DOI:** 10.3390/insects12060482

**Published:** 2021-05-21

**Authors:** Wigunda Rattanapun, Manop Tarasin, Suraphon Thitithanakul, Yaowaphan Sontikun

**Affiliations:** 1 Economic Plant of Surat Thani Province Research Unit, Faculty of Innovative Agriculture and Fishery Establishment Project, Prince of Songkla University, Surat Thani Campus, Surat Thani 84000, Thailand; thiti43@hotmail.com (S.T.); yaowaphan.s@psu.ac.th (Y.S.); 2 Economic Plant of Surat Thani Province Research Unit, Faculty of Science and Industrial Technology, Prince of Songkla University, Surat Thani Campus, Surat Thani 84000, Thailand; manop8899@gmail.com

**Keywords:** eggplant, *Capsicum*, capsaicin, chili, pepper

## Abstract

**Simple Summary:**

*Bactrocera latifrons* (Diptera: Tephritidae) is the major pest in economic chili and eggplant of Thailand. Although *B. latifrons* has many host plant species, it does not unequally prefer each host plant. Results of this study indicated that among seven host plant species of family Solanaceae, *Capsicum* fruits were preferred by *B. latifrons* for oviposition over *Solanum* fruits. Bird chili and banana pepper were the most preferred hosts for *B. latifrons*. Turkey berry was least preferred by *B. latifrons*. Results of this study develop knowledge of the specific mechanisms by which host fruit varieties, host fruit species and physiological changes during ripening of host fruit influence *B. latifrons*’ preference. This is of potential value in field management, quarantine and plant breeding using traditional or transgenic approaches.

**Abstract:**

Host preference of *Bactrocera latifrons* (Hendel) (Diptera: Tephritidae), major chili and nightshade pest, was studied using seven host plant species of family Solanaceae. Two nightshade species, eggplant, *Solanum melongena* L. and turkey berry, *Solanum torvum* Sw.; three pepper and one large chili cultivar of *Capsicum annum* L., banana pepper, cayenne pepper, noom pepper and duey kai chili; and one small chili cultivar of *Capsicum frutescens* L., bird chili, were used as tested host plants of *B. latifrons* for a series of choice test and no-choice test under the laboratory. Results revealed that *B. latifrons* preferred *Capsicum* fruits for oviposition rather than *Solanum* fruits. Bird chili and banana pepper were the most preferred host for *B. latifrons*, with the highest number of pupae per gram of fruit in no-choice and choice experiment, respectively. Although the best larval performance parameters of *B. latifrons* were better for eggplant than for other Solanaceous plants, fruit characteristics and total phenolic content in fruit play a major role for host preference of *B. latifrons*. Turkey berry was least preferred by *B. latifrons*, with the lowest number of pupae per fruit and it was not oviposited by *B. latifrons* female fly under the choice situation at all stages of ripeness.

## 1. Introduction

Tropical fruit flies (Diptera: Tephritidae: Dacinae) are serious pests of economic fruits in Southeast Asia and the Pacific. Dacine fruit flies attack a wide range of fruit species and cause significant economic losses [1]. Some fruit fly species show a distinct preference for certain hosts when these are available, but will infest other hosts when the preferred hosts are unavailable [2]. For example, *Bactrocera jarvisi* (Tryon) (Diptera: Tephritidae) prefers the cocky apple, *Planchonia careya* (Lecythidaceae), both in the field and in laboratory experiments, and it infests this host almost exclusively when available, despite having been recorded as infesting many other fruits [3]. However, host preference of tephritid fruit fly varies in different regions. *Psidium guajava* (Myrtaceae) is the host most favored by the polyphagous fruit fly *Bactrocera dorsalis* (Hendel) (Diptera: Tephritidae) complex [4,5]. Meanwhile, other fruits such as mango and starfruit are also favored in field samplings [6,7].

*Bactrocera latifrons* was earlier distributed in Asia and the Pacific, but its range has expanded to include Hawaii, Okinawa (Japan), Tanzania and Kenya [8,9]. *Bactrocera latifrons* has a wide range of host plants, with 59 plant species of 14 plant families, predominately belonging to Solanaceae and Cucurbitaceae [10]. The adult *B. latifrons* female lays eggs in the host fruit. The larvae feed inside the fruit, causing it to rot and fall. Economic losses caused by *B. latifrons* infestations of commercial crops have been reported: for example, in Malaysia *B. latifrons* caused 60–80% infestation of red pepper crop [11]. The damage to tomato in all geographical regions of Mizoram, India, ranged from 3.6 to 28.6%, caused by *B. latifrons*, *Bactrocera correcta* (Bezzi) (Diptera: Tephritidae) and *B. dorsalis* [12].

The infestation rate of *B. latifrons* is higher in solanaceous hosts than in cucurbitaceous hosts. The authors of [13,14] reported that among the 11 solanaceous and four cucurbitaceous plants, *B. latifrons* most prefers to infest solanaceous plants: *Lycopersicon pimpinellifolium* (Jusl.) Mill., *Solanum nigrescens* Mart. & Galeotti, *Solanum sodomeum* L. and *Solanum torvum* Sw., grown in feral habitats; and three economic species of solanaceous plants in backyard and commercial cultivation, namely *Capsicum annuum* L., *Lycopersicon lycopersicum* (L.) Karst. ex Farw. and *Solanum melongena* L. A study on oviposition preference of *B. latifrons* on two wild solanaceous fruit species (*Solanum linnaeanum* and *S. torvum*) indicated that *B. latifrons* preferred *S. linnaeanum* over *S. torvum* and that its survival rate is higher than the former [15]. The authors of [16] reported that *B. latifrons* has a preference for mature eggplant, and the fact that eggplant is harvested at the mature green stage might eliminate the need for quarantine restrictions. The authors of [17,18] indicated that *B. latifrons* utilizes eggplant as a host throughout its distribution. The authors of [17] indicated that the physiological stage of host fruit influences the host preference of *B. latifrons*. Green and purple eggplants that have turned yellow, being overripe, are utilized by *B. latifrons* more than by other fruit fly species. Generally, female flies use information from host fruits to determine the quality of that host fruit for offspring growth and survival, such as fruit firmness [19,20], fruit size [21] and secondary metabolic compounds in fruit [22]. Distinctly secondary metabolic compounds of plant in family Solanaceae for resistance to insect pest are phenolic compounds [23,24,25] and capsaicins [26,27,28]. These chemical compounds will be accumulated along fruit growth.

The objective of this study was to get to know the preference of *B. latifrons* for economic and non-economic host fruit varieties of family Solanaceae and the fruit physiology influence on their preferences. This is of potential value for field management and quarantine in the future.

## 2. Materials and Methods

### 2.1. Insect Preparation

*Bactrocera latifrons* used for this study originated from infested fruits of *C. annuum* L. Jinda variety, collected from a commercial chili plot in Muang district, Surat Thani province, Thailand. Male and female flies were reared in 30 × 30 × 30 cm^3^ Perspex cages. Water and a 3:1 mixture of sugar and yeast hydrolysate were provided in the cage for adult flies. *Bactrocera latifrons* mass rearing used host fruit in the experiment (family Solanaceae): two nightshade species (eggplant *S. melongena* L. and turkey berry *S. torvum* Sw.); three pepper varieties of *C. annuum* L. (banana pepper, cayenne pepper and noom pepper); one large chili variety of *C. annuum* L. (duey kai chili) and a small chili variety of *Capsicum frutescens* L. (bird chili). All the fruits were in unripe stage (18 days after full bloom for eggplant and turkey berry, 25, 26, 28, 28 and 39 days after full bloom for duey kai chili, banana pepper, noom pepper, cayenne pepper and bird chili, respectively). Average humidity, temperature, light intensity and photoperiod within the laboratory were 65%, 25 °C, 330 Lux of neon light and L12:D12 h, respectively. F2 and F3 male and gravid female offspring were used in the experiment.

### 2.2. Host Fruit Preparation

The solanaceous fruits used for *B. latifrons* rearing were grown in plant pots (25 cm diameter, 40 cm height). All the pots were kept in an 85 × 85 × 130 cm^3^ cage covered with a mesh for pest protection. Fruits of each species were collected for *B. latifrons* mass rearing and experiments. The number of days after full bloom in unripe fruits used for the experiment was the same as that of unripe fruit used for *B. latifrons* mass rearing. For ripe fruits, it was 30 days after full bloom fruit for eggplant and turkey berry and 35, 46, 49, 60 and 60 days after full bloom for duey kai chili, banana pepper, bird chili, noom and cayenne pepper, respectively.

#### 2.2.1. No-Choice Experiment

For host fruit preference of *B. latifrons* under the no-choice situation, five pairs of 20–22-day-old male and female flies of *B. latifrons* were placed in the 30 × 30 × 30 cm^3^ metal cage, along with five fruits of one solanaceous plant species or variety. The fruits were randomly collected for the experiment. Fruit stem was covered with moisture cotton and wrapped with tin foil for keeping the fruit fresh. All the fruits were hung from the top of the observation cage, with a string at the fruit stem. Fruit spacing was four cm. Flies were left in the metal cage for three days with water and a 3:1 mixture of sugar and yeast hydrolysate. Five replicates were conducted for unripe and ripe stage of each solanaceous plant species. After three days, all the fruits were collected from the cage and incubated in separate 20 × 26 × 12 cm^3^ plastic containers with sand for 15–16 days until the larvae had pupated. At the end of the experiment, females were dissected to check if eggs were present in their ovaries. The pupae were collected every three days until absence of pupae in sand. Number of pupae was recorded. All pupae were weighted using an electronic analytical balance (ATY 224, Shimadzu, Co., Kyoto, Japan) and left in small plastic bottles for adult eclosion. The number of emerged adults was recorded.

#### 2.2.2. Choice Experiment

The oviposition preference for host fruit species of *B. latifrons* was tested under the choice situation when one fruit of each tested solanaceous plant was offered simultaneously with the others in a 30 × 30 × 30 cm^3^ metal cage. With the exception of simultaneous offering of fruits, the rest of the experimental conditions were as in the no-choice trial. Five replicates were conducted for unripe and ripe stage of the fruits.

### 2.3. Fruit Characteristics

Separately from the fruits used in actual trials, ten fruits from each ripening stage of the solanaceous plant species and varieties were randomly selected for measurements of fruit width and length using vernier caliper (500-196-30, Mitutoyo, Japan). Three unripe and ripe fruits were measured for fruit firmness using a fruit pressure tester (FT-011, Effegi, Italy). Two secondary metabolic compounds, total phenolic content and capsaicin were measured. Total phenolic content plays important roles in the resistance mechanism of plants against insect [23,24,25] while capsaicin can deter many insects and causes larval deaths [26,27,28]. The concentration of total phenolic content and capsaicin constituents of solanaceous fruits varies with ripening stage and varieties or species [29,30,31]. Total phenolic content was measured using the colorimetric method with Folin–Ciocalteu reagent [32], and gallic acid was used as the standard. Capsaicin content was analyzed using the technique of [33] for capsaicinoid extraction and capsaicin content analysis was conducted using high performance liquid chromatography (HPLC) (Shimadzu LC-10 Series, Shimadzu, CO., Kyoto, Japan).

### 2.4. Statistical Analyses

In order to compare differences among fruit species or varieties, data (pupae number and pupae weight, average weight of one pupa, percentage of adult emergence, number of offspring per fruit and fruit physical characteristics of solanaceous plants) were analyzed by analysis of variance (ANOVA) followed by multiple comparison of means using Tukey’s honestly significant difference (HSD). Paired-sample t-test was used to analyze the difference among the number of male and female emerged offspring. The significance level for all analyses was 0.05. The data were transformed, if required, to meet the assumptions of ANOVA, and then back-transformed for shown values in tables. The data were analyzed using SPSS statistics version 17.0.

## 3. Results

### 3.1. Host Preference of Bactrocera Latifrons on Unripe Solanaceous Fruits in a No-Choice Experiment

The host preference testing of *B. latifrons* on unripe solanaceous fruits in a no-choice situation showed that there were significant differences in the number of pupae per gram of fruit (ANOVA: number of pupae/gram of fruit F_6,28_ = 16.502, *p* ≤ 0.0001) (Table 1). Number of pupae per gram of fruit was highest in bird chili, followed by cayenne pepper, duey kai chili and banana pepper, respectively. Eggplant had the lowest number of pupae per gram of fruit. Results of the larval performance factors (total weight of pupae per fruit, average weight of one pupa, percentage of adult emergence and number of offspring per fruit) showed that total weight of pupae per fruit and number of offspring per fruit of *B. latifrons* in eggplant was significantly higher than that of all other solanaceous plants. The percentage of adult emergence was highest in eggplant. There were no significant differences in the percentage of adult emergence among eggplant, cayenne pepper, noom pepper, bird chili and banana pepper. Average weight of one pupa was highest in eggplant and not significantly different with cayenne pepper and noom pepper (ANOVA: total weight of pupae/fruit F_6,24_ = 11.457, *p* ≤ 0.0001; average weight of one pupa F_6,24_ = 5.430, *p* = 0.001; percentage of adult emergence F_6,24_ = 4.869, *p* = 0.002; number of offspring/fruit F_6,24_ = 14.273, *p* ≤0.0001) (Table 1). In addition, turkey berry was the least suitable host for *B. latifrons* compared with other solanaceous fruits. With the exception of eggplant and duey kai chili, almost all of solanaceous plants showed 1:1 (male:female) sex ratio of *B. latifrons* (paired-sample *t*-test: eggplant *t* = 4.140, *p* = 0.001; banana pepper *t* = 0.743, *p* = 0.466; cayenne pepper *t* = −1.914, *p* = 0.072; noom pepper *t* = −0.316, *p* = 0.760; bird chili *t* = 0.822, *p* = 0.426; duey kai chili *t* = 1.158, *p* = 0.285) (Table 1).

### 3.2. Host Preference of Bactrocera Latifrons on Ripe Solanaceous Fruits in a No-Choice Experiment

Similarly, among ripe fruit, number of pupae per gram of fruit was highest in bird chili over the others for *B. latifrons* oviposition in the no-choice experiment, followed by duey kai chili, turkey berry, cayenne pepper, noom pepper and banana pepper, respectively. Again, eggplant had the lowest number of pupae per gram of fruit (ANOVA: number of pupae/gram of fruit F_6,28_ = 6.322, *p* ≤ 0.0001) (Table 2). Results revealed that with the exception of percentage of adult emergence, all larval performance factors of *B. latifrons* were highest in ripe eggplant fruit and it was not significantly different with banana pepper and noom pepper. In addition, bird chili had the highest percentage of adult emergence and it was not significantly different with eggplant. The larval performance factors of *B. latifrons* in the remaining solanaceous plant were not significantly different (ANOVA: total weight of pupae/fruit F_6,26_ = 10.156, *p* ≤ 0.0001; average weight of one pupa F_6,26_ = 3.402, *p* = 0.013; number of offspring/fruit F_6,27_ = 7.025, *p* ≤ 0.0001; percentage of adult emergence F_6,26_ = 2.494, *p* = 0.049). Result of sex ratio in ripe fruit presented that, unless bird chili, 1:1 (male:female) sex ratio of *B. latifrons* was found in almost all solanaceous plants (paired-sample *t*-test: eggplant *t* = 1.635, *p* = 0.115; turkey berry *t* = −0.329, *p* = 0.756; banana pepper *t* = −0.909, *p* = 0.378; cayenne pepper *t* = 1.012, *p* = 0.328; noom pepper *t* = 2.291, *p* = 0.034; bird chili *t* = 4.696, *p* ≤ 0.0001) (Table 2).

### 3.3. Host Preference of Bactrocera Latifrons on Solanaceous Fruits in a Choice Experiment

Results from ANOVA (split-plot design) identified that under the choice situation, there were no significant interaction effects between ripening stage and plant species in the number of pupae per gram of fruit (ANOVA: F_6,24_ = 0.911, *p* = 0.504), total weight of pupae per fruit (F_3,6_ = 2.484, *p* = 0.158), average weight of one pupa (ANOVA: F_3,6_ = 1.609, *p* = 0.284), percentage of adult emergence (ANOVA: F_3,6_ = 2.089, *p* = 0.203) and number of offspring per fruit (ANOVA: F_3,6_ = 2.085, *p* = 0.204). There were highly significant differences among all parameters of larval performance when data were pooled across ripening stage and compared between plant species (Table 3). Results of the choice experiment present a clear host fruit preference and host suitability of *B. latifrons*. Under the choice situation, *B. latifrons* most highly preferred banana pepper for oviposition with the highest number of pupae per gram of fruit, followed by three pepper species, bird chili, noom pepper and cayenne pepper. In addition, number of pupae per gram of fruit was higher in eggplant than that of duey kai chili (Table 3). Only one infested fruit of duey kai chili was found. Interestingly, there were no fruits of turkey berry oviposited under the choice situation. There were no significant differences in the average weight of one pupa and percentage of adult emergence among eggplant, banana pepper, cayenne pepper, noom pepper and bird chili. Total weight of pupae per fruit and number of offspring per fruit in eggplant were significantly higher than those of all other solanaceous plants (Table 3). With the exception of banana pepper and cayenne pepper, *B. latifrons* had 1:1 (male:female) sex ratio in almost all solanaceous plants (paired-sample t-test: eggplant t = 0.285, *p* = 0.784; banana pepper t = 2.321, *p* = 0.049; cayenne pepper t = 4.981, *p* = 0.004; noom pepper t = 1.158, *p* = 0.311; bird chili t = 0.408, *p* = 0.704).

Eggplant, noom pepper and banana pepper were bigger than the other solanaceous fruits (one-way ANOVA: unripe fruit width: F_6,63_ = 338.921, *p* ≤ 0.0001; ripe fruit width: F_6,63_ = 352.190, *p* ≤ 0.0001; unripe fruit length: F_6,63_ = 333.427, *p* ≤ 0.0001; ripe fruit length: F_6,63_ = 422.086, *p* ≤ 0.0001) (Table 4). Moreover, the mean firmness differed significantly between the solanaceous fruits (one-way ANOVA: unripe fruit F_6,14_ = 19.399, *p* ≤ 0.0001; ripe fruit F_6,14_ = 57.188, *p* ≤ 0.0001). Eggplant had a tougher exopericarp than the other solanaceous fruits, followed by turkey berry, while banana pepper was the least firm. With the exception of eggplant and turkey berry, fruit size increased but firmness decreased with ripening (Table 4).

Regarding the total phenolic content, there were significant differences in the total phenolic content among the solanaceous plants at any stage of ripeness (one-way ANOVA: unripe fruit: F_6,14_ = 557.801, *p* ≤ 0.0001; ripe fruit: F_6,14_ = 154.813, *p* ≤ 0.0001) (Table 5). *Solanum* plants had significantly higher contents than *Capsicum* plants in all ripening stages. Turkey berry had the highest total phenolic content, followed by eggplant. Total phenolic content was not a significant difference among five varieties of *Capsicum* plants. Regarding capsaicin in unripe fruit, there were significant differences in capsaicin quantities in unripe fruit among the five varieties of *Capsicum* plant. Bird chili and duey kai chili had the most capsaicin content, followed by cayenne pepper, banana pepper and noom pepper, respectively. For ripe fruit, ripe bird chili had the most capsaicin content, followed by duey kai chili, and no significant difference was found among cayenne pepper and noom pepper. Ripe banana pepper had the least capsaicin content (one-way ANOVA: unripe fruit: F_4,10_ = 686.767, *p* ≤ 0.0001; ripe fruit: F_4,10_ = 1483.352, *p* ≤ 0.0001) (Table 5).

## 4. Discussion

*Bactrocera latifrons* has diverse host plant species in the family Solanaceae, but it does show definite preferences among various host plants. The study revealed that *B. latifrons* preferred *Capsicum* fruits rather than *Solanum* fruits for oviposition. Bird chili and banana pepper were the most preferred host for oviposition by *B. latifrons*, with the highest number of pupae per gram of fruit under the no-choice and choice experiment, respectively. In addition, cayenne pepper tended to be the preferred host of *B. latifrons*, also with a high number of pupae per gram of fruit in both no-choice and choice experiment. Although eggplant was the least preferred by *B. latifrons* under the no-choice experiment, for choice experiment duey kai chili and turkey berry were the least preferred host with only one pupae and complete absence of pupae, respectively. Generally, the oviposition choice of female flies is strongly related to maximizing the performance of their offspring [15,34,35] and also to fruit firmness, allowing easier oviposition [18,36]. Results of this study indicated that both fruit characteristics and some plant secondary compounds influenced *B. latifrons* preference. The fruit characteristics indicate that *Capsicum* fruits were softer than *Solanum* fruits and with lower total phenolic content; plant secondary metabolites play important roles in plant resistance [23,24,25] at all ripening stages. Hence, *B. latifrons* female flies preferred *Capsicum* fruits for oviposition over *Solanum* fruits. Moreover, results of this study indicate that capsaicin was not a major factor that influenced preference of *B. latifrons* between different ripening stages of fruit with higher numbers of infested fruit and offspring in ripe fruit over unripe fruit of *Capsicum* plant in the no-choice experiment. Moreover, under the no-choice experiment, bird chili in all ripening stages was most preferred by *B. latifrons* for oviposition, whereas bird chili had higher capsaicin content than that of all *Capsicum* fruit. High percentage of *B. latifrons* adult emergence in bird chili may partially explain the preference of *B. latifrons* female for oviposition in bird chili. Moreover, bird chili had a slightly softer pericarp than noom pepper, duey kai chili and cayenne pepper in unripe stage. The preference of *B. latifrons* for banana pepper seemed to depend on fruit characteristics with most soft pericarp. Many tephritid species prefer to lay their eggs in soft spots or existing wounds in fruit [20,37]. Moreover, with the exception of eggplant, banana pepper was distinctly larger than the fruits of other *Capsicum* plants. Previous studies have reported that females of some tephritid fruit fly species tend to deposit larger clutches in larger hosts [19,38,39]. In addition, percentage of adult emergence and some larval performance factors of *B. latifrons* in banana pepper were not significantly different from eggplant and bird chili. Although almost all larval performance factors were better for eggplant fruit than for the other solanaceous fruits, softer pericarp and lower total phenolic compounds in *Capsicum* fruit play an important role for oviposition preference of *B. latifrons* for *Capsicum* fruit over *Solanum* fruit. Prior studies reported the most preference of *B. latifrons* for *Solanum* plant, in terms of infestation rate [17,18]. However, results of this study indicated that under the laboratory conditions, *B. latifrons* preferred *Capsicum* fruit rather than *Solanum* fruit for oviposition.

Thus, fruit firmness and total phenolic content in the host fruit affect the choices by *B. latifrons* female flies. This study also found that, even though turkey berry was not infested by *B. latifrons* in the choice experiment, adult flies were found in ripe and unripe turkey berry fruit in the no-choice experiment. Small fruit, hard pericarp and highest total phenolic content of turkey berry made it unattractive to *B. latifrons* female flies. However, turkey berry can act as an alternative host that sustains *B. latifrons* population in nature.

## 5. Conclusions

Results of this study indicated that among seven tested host plants in the family Solanaceae, bird chili and banana pepper were the most preferred tested host plants of *B. latifrons*. Our further study will be focused on the utilization of bird chili or banana pepper, as a trap crop plant, which diverts *B. latifrons* from *Capsicum* plant species (main crop) and thus reduces the damage caused by *B. latifrons*.

## Figures and Tables

**Table 1:** The mean number and weight (± SE) of pupae and adults of *Bactrocera latifrons* in the unripe fruit with no-choice experiment.

Plant’s Common Name	No. Pupae/ Gram of Fruit (Pupa)	Total Weight of Pupae/Fruit (g)	Average Weight of One Pupa (g)	Adult Emergence (%)	No. Offspring/Fruit (indiv.)	Sex Ratio Male:Female
eggplant	0.09 ± 0.01 _c_	0.1740 ± 0.0282 _a_	0.0414 ± 0.0103 _a_	78.56 ± 6.31 _a_	5.00 ± 0.67 _a_	3:1
n	5	5	5	5	5	20
turkey berry	0.70 ± 0.11 _b_	0.0071 ± 0.0008 _b_	0.0062 ± 0.0031 _b_	18.75 ± 13.52 _c_	0.33 ± 0.33 _c_	-
n	5	3	3	3	3	-
banana pepper	0.88 ± 0.14 _b_	0.0306 ± 0.0034 _b_	0.0114 ± 0.0008 _b_	57.35 ± 6.72 _ab_	1.60 ± 0.29 _b_	1:1
n	5	5	5	5	5	21
cayenne pepper	1.22 ± 0.28 _ab_	0.0629 ± 0.0327 _b_	0.0207 ± 0.0075 _ab_	71.75 ± 7.48 _ab_	1.83 ± 0.46 _b_	1:1
n	5	5	5	5	5	19
noom pepper	0.10 ± 0.02 _c_	0.0225 ± 0.0040 _b_	0.0158 ± 0.0012 _ab_	70.04 ± 8.69 _ab_	1.13 ± 0.13 _bc_	1:1
n	5	4	4	4	4	9
bird chili	1.78 ± 0.25 _a_	0.0164 ± 0.0023 _b_	0.0106 ± 0.0012 _b_	62.04 ± 9.60 _ab_	0.93 ± 0.12 _bc_	1:1
n	5	5	5	5	5	14
duey kai chili	1.22 ± 0.27 _ab_	0.0060 ± 0.0006 _b_	0.0041 ± 0.0005 _b_	41.67 ± 11.56 _bc_	0.60 ± 0.16 _bc_	2:1
n	5	4	4	4	4	

With the exception of sex ratio, n = the number of replicated cohorts. With the exception of the number of pupae per gram of fruit, uninfested fruit (pupae were not found) was not used as replicate in other parameters. Sex ratio samples were the number of infested fruit with emerged *B. latifrons* offspring. Sex ratio of *B. latifrons* in turkey berry was not analyzed because two turkey berry fruits had one male and one female of each fruit. Values (mean ± SE) in the same column followed by different letters are statistically different (Tukey test, *p* < 0.05). Significance is based on log (x + 1) transformed data, but non-transformed data are presented.

**Table 2:** The mean number and weight (± SE) of pupae and adults of *Bactrocera latifrons* in ripe fruit of the no-choice experiment.

Plant’s Common Name	No. Pupae/Gram of Fruit (Pupa)	Total Weight of Pupae/Fruit (g)	Average Weight of One Pupa (g)	Adult Emergence (%)	No. Offspring/Fruit (indiv)	Sex Ratio Male:Female
eggplant	0.09 ± 0.01 _c_	0.1746 ± 0.0323 _a_	0.0258 ± 0.0062 _a_	89.48 ± 4.86 _a_	6.72 ± 0.90 _a_	1:1
n	5	5	5	5	5	25
turkey berry	1.15 ± 0.51 _abc_	0.0208 ± 0.0087 _c_	0.0087 ± 0.0013 _b_	40.63 ± 10.81 _b_	0.85 ± 0.17 _c_	1:1
n	5	4	4	4	4	6
banana pepper	0.45 ± 0.11 _c_	0.0955 ± 0.0182 _abc_	0.0177 ± 0.0018 _ab_	61.19 ± 11.02 _ab_	3.84 ± 1.02 _ab_	1:1
n	5	5	5	5	5	16
cayenne pepper	1.02 ± 0.28 _abc_	0.0604 ± 0.0090 _bc_	0.0159 ± 0.0004 _ab_	60.45 ± 13.03 _ab_	2.13 ± 0.44 _bc_	1:1
n	5	5	5	5	5	16
noom pepper	0.58 ± 0.14 _bc_	0.1022 ± 0.0174 _ab_	0.0162 ± 0.0013 _ab_	65.28 ± 9.11 _ab_	4.00 ± 0.54 _ab_	1:1
n	5	5	5	5	5	20
bird chili	3.23 ± 1.00 _a_	0.0332 ± 0.0138 _bc_	0.0128 ± 0.0040 _ab_	90.63 ± 4.38 _a_	2.17 ± 0.36 _bc_	4:1
n	5	4	4	4	4	16
duey kai chili	2.34 ± 0.31 _ab_	0.0285 ± 0.0050 _bc_	0.0109 ± 0.0015 _b_	72.50 ± 12.75 _ab_	2.03 ± 0.46 _bc_	1:1
n	5	5	5	5	5	

With the exception of sex ratio, n = the number of replicated cohorts. With the exception of the number of pupae per gram of fruit, uninfested fruit (pupae were not found) was not used as replicate in other parameters. Sex ratio samples were number of infested fruit with emerged *B. latifrons* offspring. Values (mean ± SE) in the same column followed by different letters are statistically different (Tukey test, *p* < 0.05). Significance is based on log (x + 1) transformed data, but non-transformed data are presented.

**Table 3:** The mean number and weight (± SE) of pupae and adults of *Bactrocera latifrons* in each plant species of all ripening stages of the choice experiment.

Plant’s Common Name	No. Pupae/ Gram of Fruit (Pupa)	Total Weight of Pupae/Fruit (g)	Average Weight of One Pupa (g)	Adult Emergence (%)	No. Offspring/Fruit (indiv)	Sex Ratio Male:Female
eggplant	0.27± 0.05 _abc_	0.3309± 0.0688 _a_	0.0164 ± 0.0008	73.83 ± 9.63	16.00 ± 3.61 _a_	1:1
n	10	9	9	9	9	8
turkey berry	0.00± 0.00 _c_	-	-	-	-	-
n	10	-	-	-	-	-
banana pepper	0.80± 0.14 _a_	0.0943± 0.0153 _b_	0.0141± 0.0006	74.95 ± 10.13	4.44 ± 0.82 _bc_	2:1
n	10	9	9	9	9	9
cayenne pepper	0.60± 0.18 _ab_	0.0294± 0.0056 _b_	0.0183 ± 0.0029	80.95 ± 14.29	1.43 ± 0.37 _cd_	10:1
n	10	7	7	7	7	6
noom pepper	0.61± 0.07 _ab_	0.0906± 0.0126 _b_	0.0155 ± 0.0005	79.50 ± 6.77	4.56 ± 0.60 _b_	1:1
n	10	9	9	9	9	5
bird chili	0.66± 0.23 _ab_	0.0123± 0.0011 _b_	0.0123± 0.0011	100.00 ± 0.00	1.00 ± 0.00 _d_	1:1
n	10	5	5	5	5	5
duey kai chili	0.16± 0.16 _b__c_	0.0052	0.0052	100.00	1	-
n	10	1	1	1	1	-
F-test	F_6,__63_ = 5.242, *p* ≤ 0.0001	F_4,20.221_ = 12.470, *p* ≤ 0.0001	F_4,16.875_ = 2.472, *p* = 0.084	F_4,18.237_ = 1.136, *p* = 0.371	F_4,16.922_ = 4.583, *p* = 0.011	

With the exception of sex ratio, n = the number of replicated cohorts. With the exception of the number of pupae per gram of fruit, uninfested fruit (pupae were not found) was not used as replicate in other parameters. Sex ratio samples were number of infested fruit with emerged *B. latifrons* offspring. Values (mean ± SE) in the same column followed by different letters are statistically different (Tukey test, *p* < 0.05). Significance is based on log (x + 1) transformed data, while non-transformed data are presented.

**Table 4:** Fruit characteristics of seven solanaceous plants used in the experiment.

Plant’s Common Name	Fruit Width (mm)		Fruit Length (mm)		Fruit Firmness (kg/m^2^)	
	Unripe n = 10	Ripe n = 10	Unripe n = 10	Ripe n = 10	Unripe n = 3	Ripe n = 3
eggplant	44.82 ± 2.39 _a_	47.99 ± 2.39 _a_	51.23 ± 1.59 _c_	52.56 ± 2.27 _c_	1.55 ± 0.18 _a_	1.62 ± 0.16 _a_
turkey berry	10.07 ± 0.41 _c_	11.17 ± 0.13 _d_	12.21 ± 0.26 _e_	12.11 ± 0.19 _g_	1.02 ± 0.06 _b_	1.07 ± 0.03 _b_
banana pepper	26.21 ± 1.11 _b_	31.39 ± 1.24 _b_	96.25 ± 3.38 _b_	101.07 ± 4.15 _b_	0.53 ± 0.04 _d_	0.42 ± 0.02 _d_
cayenne pepper	10.27 ± 0.40 _c_	10.74 ± 0.51 _d_	42.94 ± 2.99 _c_	44.02 ± 2.50 _d_	0.73 ± 0.04 _bcd_	0.50 ± 0.00 _cd_
noom pepper	26.46 ± 0.82 _b_	21.71 ± 1.01 _c_	148.62 ± 2.71 _a_	147.39 ± 2.61 _a_	0.88 ± 0.04 _bc_	0.72 ± 0.02 _c_
bird chili	6.19 ± 0.20 _d_	6.84 ± 0.16 _e_	25.01 ± 0.88 _d_	26.04 ± 1.23 _f_	0.68 ± 0.06 _cd_	0.68 ± 0.04 _c_
duey kai chili	5.32 ± 0.36 _d_	5.52 ± 0.35 _f_	29.54 ± 1.97 _d_	34.98 ± 1.31 _e_	0.82 ± 0.02 _bcd_	0.53 ± 0.03 _cd_

n = number of replicates. Values (mean ± SE) in the same column followed by different letters are statistically different (Tukey test, *p* <0.05). Non-transformed data are presented.

**Table 5:** Capsaicin and total phenolic contents found in seven solanaceous fruits.

Plant’s Common Name	Total Phenolic Content (mgGAE/g FW)		Capsaicin (mg/g DW)	
	Unripe (n = 3)	Ripe (n = 3)	Unripe (n = 3)	Ripe (n = 3)
eggplant	3.90 ± 0.07 _b_	2.72 ± 0.24 _b_	-	-
turkey berry	6.80 ± 0.16 _a_	6.48 ± 0.24 _a_	-	-
banana pepper	1.35 ± 0.06 _c_	1.68 ± 0.08 _c_	0.66 ± 0.02 _d_	1.30 ± 0.02 _d_
cayenne pepper	1.48 ± 0.09 _c_	1.91 ± 0.01 _c_	1.43 ± 0.04 _b_	1.63 ± 0.03 _c_
noom pepper	1.23 ± 0.04 _c_	1.63 ± 0.08 _c_	0.85 ± 0.02 _c_	1.35 ± 0.02 _cd_
bird chili	1.13 ± 0.04 _c_	1.61 ± 0.11 _c_	2.55 ± 0.04 _a_	3.31 ± 0.02 _a_
duey kai chili	1.33 ± 0.11 _c_	1.84 ± 0.06 _c_	2.54 ± 0.04 _a_	2.85 ± 0.03 _b_

n = number of replicates. Values (mean ± SE) in the same column followed by different letters are statistically different (Tukey test, *p* < 0.05). Non-transformed data are presented.

## Data Availability

All datasets supporting the conclusion of this article are included in the article. Data will not be shared in any other source.
